# p38/AP-1 Pathway in Lipopolysaccharide-Induced Inflammatory Responses Is Negatively Modulated by Electrical Stimulation

**DOI:** 10.1155/2013/183042

**Published:** 2013-04-14

**Authors:** Deok Jeong, Jaehwi Lee, Young-Su Yi, Yanyan Yang, Kyoung Won Kim, Jae Youl Cho

**Affiliations:** ^1^Department of Genetic Engineering, Sungkyunkwan University, Suwon 440-746, Republic of Korea; ^2^College of Pharmacy, Chung-Ang University, Seoul 156-756, Republic of Korea; ^3^Research Institute, Human Nanoelectrotech Co. Ltd., Seoul 151-050, Republic of Korea

## Abstract

Electrical stimulation with a weak current has been demonstrated to modulate various cellular and physiological responses, including the differentiation of mesenchymal stem cells and acute or chronic physical pain. Thus, a variety of investigations regarding the physiological role of nano- or microlevel currents at the cellular level are actively proceeding in the field of alternative medicine. In this study, we focused on the anti-inflammatory activity of aluminum-copper patches (ACPs) under macrophage-mediated inflammatory conditions. ACPs generated nanolevel currents ranging from 30 to 55 nA in solution conditions. Interestingly, the nanocurrent-generating aluminum-copper patches (NGACPs) were able to suppress both lipopolysaccharide-(LPS-) and pam3CSK-induced inflammatory responses such as NO and PGE_2_ production in both RAW264.7 cells and peritoneal macrophages at the transcriptional level. Through immunoblotting and immunoprecipitation analyses, we found that p38/AP-1 could be the major inhibitory pathway in the NGACP-mediated anti-inflammatory response. Indeed, inhibition of p38 by SB203580 showed similar inhibitory activity of the production of TNF-**α** and PGE_2_ and the expression of TNF-**α** and COX-2 mRNA. These results suggest that ACP-induced nanocurrents alter signal transduction pathways that are involved in the inflammatory response and could therefore be utilized in the treatment of various inflammatory diseases such as arthritis and colitis.

## 1. Introduction

Inflammation is a basic signal that prompts the body to clear infecting or invading pathogens from our tissues. This response is naturally present at birth; thus, it is classified as an innate immune response [1, 2]. Phagocytes such as macrophages and dendritic cells are the principal cells that manage the inflammatory response. Currently, numerous cellular events involved in inflammation have been identified. Thus, many types of soluble factors, including cytokines (e.g., tumor necrosis factor (TNF)-*α*), chemokines, and inflammatory mediators (including nitric oxide (NO) and prostaglandin E_2_ (PGE_2_)), control autocrine and paracrine types of immune cell activation, cell-to-cell interactions, and the migration of various immune cells, including macrophages, neutrophils, and T and B lymphocytes. Despite the positive role of inflammation, hyperactive and long-lasting inflammatory states are critically linked to the onset of various serious diseases, including cancer, diabetes, atherosclerosis, and arthritis [3–6]. Therefore, returning upregulated chronic inflammatory responses to baseline levels could be an important strategy to prevent multiple diseases.

The molecular aspects of the inflammatory response are principally studied in macrophages by activating pattern recognition receptors such as Toll-like receptor (TLR) 4 and TLR2 through treatment with their ligands, lipopolysaccharide (LPS) and poly(I:C), respectively [7]. Over the course of many studies, multiple intracellular signaling pathways that are composed of nonreceptor protein tyrosine kinases and mitogen-activated protein kinases (MAPKs), including ERK (extracellular signal-related kinase), p38, and JNK (C-Jun N-terminal kinase), have been identified as the upstream machinery for the activation and translocation of transcription factors (e.g., nuclear factor (NF)-*κ*B and activator protein (AP)-1) into the nucleus [8, 9]. Finally, proinflammatory genes encoding tumor necrosis factor (TNF)-*α*, inducible NO synthase (iNOS), and cyclooxygenase (COX)-2 are transcribed by these factors in activated macrophages [3, 10–12].

Various forms of radiation, including far-infrared and ultraviolet radiation, have been shown to modulate cellular and physiological responses in many different fields [13]. For example, far-infrared radiation is widely applied to promote health, treat vascular-related disorders, and preserve food [14]. In addition, improvements in endothelial cell function, enhancements of blood circulation in the skin, and increased survival among arteriovenous-fistula hemodialysis patients have also been demonstrated through the use of this radiation [15, 16]. Compared with these reports, the use of electrical stimulation is limited due to side effects. The most popular research area for electrical stimulation is the search for a method to rehabilitate neural degeneration after spinal cord injury [17]. Furthermore, acute and chronic pain and the inflammatory response are conditions that electrical stimulation has recently been employed to relieve [18–20]. Moreover, it has been reported that a weak direct current that is generated using metals such as silver at the positive electrode is able to suppress the growth of bacteria and fungi [21, 22]. Although a variety of curative methods that use microcurrents have been employed against many different types of diseases, the ameliorative mechanism of electrical stimulation has not been fully elucidated at the molecular level. Therefore, in this study, we aimed to understand the mechanisms through which electrical stimulation is able to suppress inflammatory responses at the cellular and molecular levels using a nanocurrent-generating aluminum-copper patch (NGACP). 

## 2. Materials and Methods

### 2.1. Materials

The nanocurrent-generating aluminum-copper patch (NGACP) was obtained from Human Nano Electrotech (Seoul, Korea). Pam3CSK; 3-(4,5-dimethylthiazol-2-yl)-2,5-diphenyltetrazolium bromide, a tetrazole (MTT); and lipopolysaccharide (LPS, *E. coli* 0111 : B4) were purchased from Sigma Chemical Co. (St. Louis, MO, USA). SB203580 was obtained from Calbiochem (La Jolla, CA, USA). Enzyme immunoassay (EIA) kits and enzyme-linked immunosorbent assay (ELISA) kits for determining PGE_2_ and TNF-*α* levels were purchased from Amersham (Little Chalfont, Buckinghamshire, UK). Fetal bovine serum and RPMI 1640 were obtained from GIBCO (Grand Island, NY, USA). RAW264.7 and HEK293 cells were procured from the ATCC (Rockville, MD, USA). All other chemicals were of analytical grade and obtained from Sigma. Phospho-specific or total antibodies against c-Fos, c-Jun, extracellular signal-related kinase (ERK), c-Jun N-terminal kinase (JNK), p38, mitogen-activated protein kinase kinase 3/6 (MKK3/6), MKP-1, lamin A/C, and *β*-actin were obtained from Cell Signaling (Beverly, MA, USA). 

### 2.2. Preparation of Aluminum-Copper Patch and Measurement of the Electrical Potential

Copper and aluminum patches used in the present study were obtained from a commercial source (Pharmdi Band) manufactured under the method below. Copper having a purity of 99.92% was cold-rolled to produce a thin metal plate with a thickness of 100 *µ*m and cut into a square (1 cm^2^) and used as a cathode. Separately, aluminum patch (99.70% purity, 17 *μ*m thickness, 1 cm^2^ area) was produced using the same procedure and used as an anode. For measuring electrical potential, two electrodes (cathode and anode) were attached to the main body of a multimeter (DMM 4040, Tektronix, USA). The electrodes were immersed in cell culture media or saline and placed in a polypropylene dish, and the electric current values were monitored. Separately, nanocurrent-generating aluminum and copper patches (NGACPs) with surface areas of 0.4 cm^2^ were held on the bottom surface of the dish using double-sided adhesive tape, and cell culture media or saline (10 mL) was added to the dish. Then, the cathodes and anodes were firmly touched to the aluminum and copper patches, and the electric current values were obtained. 

### 2.3. Animals

C57BL/6 male mice (6- to 8-week old, 17 to 21 g) were obtained from DAEHAN BIOLINK (Chungbuk, Korea) and housed in plastic cages under conventional conditions. Water and pellet diets (Samyang, Daejeon, Korea) were available *ad libitum*. Studies were performed in accordance with the guidelines established by the Sungkyunkwan University Institutional Animal Care and Use Committee.

### 2.4. Preparation of Peritoneal Macrophages

Peritoneal exudates were obtained from C57BL/6 male mice (7- to 8-week old, 17 to 21 g) by lavaging four days after intraperitoneal injection of 1 mL of sterile 4% thioglycolate broth (Difco Laboratories, Detroit, MI, USA) as previously reported [23]. After washing with RPMI 1640 medium containing 2% FBS, peritoneal macrophages (1 × 10^6^ cells/mL) were plated in 100 mm tissue culture dishes for 4 h at 37°C in a 5% CO_2_ humidified atmosphere.

### 2.5. Cell Culture

Peritoneal macrophages and cell lines (RAW264.7 and HEK293 cells) were cultured with RPMI 1640 medium supplemented with 10% heat-inactivated fetal bovine serum (Gibco, Grand Island, NY, USA), glutamine, and antibiotics (penicillin and streptomycin) at 37°C under 5% CO_2_. For each experiment, the cells were detached with a cell scraper. At the cell density that was used for the experiments (2 × 10^6^ cells/mL), the proportion of dead cells was less than 1% as measured by Trypan blue dye exclusion. 

### 2.6. NO, PGE_2_, and TNF-*α* Production

After recuperation of RAW264.7 cells or peritoneal macrophages (1 × 10^6^ cells/mL) for 18 h, the cells were placed with NGACPs for 30 min and were further incubated with LPS (1 *μ*g/mL) for 24 h. The inhibitory effect of NGACPs treatment on NO, PGE_2_, and TNF-*α* production was determined by analyzing NO, PGE_2_, and TNF-*α* levels with the Griess reagent and enzyme-linked immunosorbent assay (ELISA) kits as previously described [24, 25]. 

### 2.7. Cell Viability Test

After preincubation of RAW264.7 cells (1 × 10^6^ cells/mL) for 18 h, NGACPs was added to the cells, and the cells were incubated for 24 h. The cytotoxic effect of the peptide K5 was then evaluated using a conventional MTT assay as previously reported [26, 27]. At 3 h prior to culture termination, 10 *μ*L of MTT solution (10 mg/mL in phosphate buffered saline, pH 7.4) was added to each well, and the cells were continuously cultured until termination of the experiment. The incubation was halted by the addition of 15% sodium dodecyl sulfate into each well, thus solubilizing the formazan [28]. The absorbance at 570 nm (OD_570–630_) was measured using a Spectramax 250 microplate reader.

### 2.8. mRNA Analysis Using Real-Time Reverse Transcriptase Polymerase Chain Reaction (RT-PCR)

To determine the cytokine mRNA expression levels, total RNA was isolated from LPS-treated RAW264.7 cells using TRIzol Reagent (Gibco BRL) according to the manufacturer's instructions. Total RNA was stored at −70°C until use. Quantification of mRNA was also performed using real-time RT-PCR with SYBR Premix Ex Taq (Takara, Japan) and a real-time thermal cycler (Bio-Rad, Hercules, CA, USA), as stated earlier [29, 30]. The results were expressed as the ratio of the optical density to GAPDH. To qualitatively evaluate mRNA expression levels, semiquantitative RT reactions were conducted as stated in advance [31]. The primers (Bioneer, Daejeon, Korea) used are indicated in Table 1.

### 2.9. Preparation of Total Lysates and Nuclear Extracts and Immunoblotting

Preparation of total lysates and nuclear extracts from LPS-treated RAW264.7 cells that had been pretreated with NGACPs was performed using a previously published method [32]. Immunoblot analysis of the levels of phosphorylated or total transcription factors (p65, c-Jun, and c-Fos), lamin A/C, MAPKs (ERK, p38, and JNK), MKK 3/6, MKP-1, and *β*-actin was performed according to the cited technique [33, 34]. 

### 2.10. Statistical Analysis

**Figure 1:**
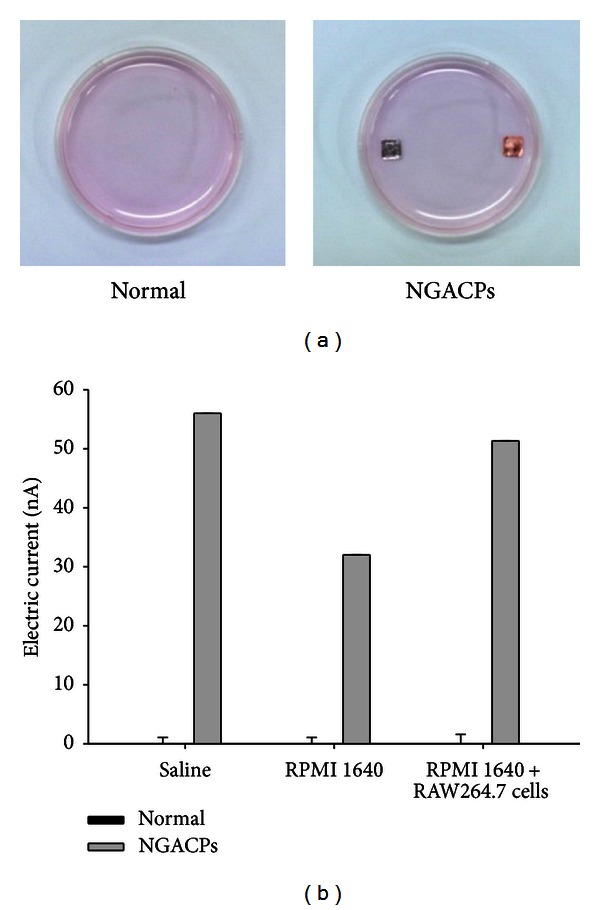
Current induction levels of aluminum-copper patch (ACP) in cell-plated medium. (a) Photos of ACPs were taken with a digital camera. (b) The electrical field was measured using a multimeter as described in Section 2.

**Figure 2:**
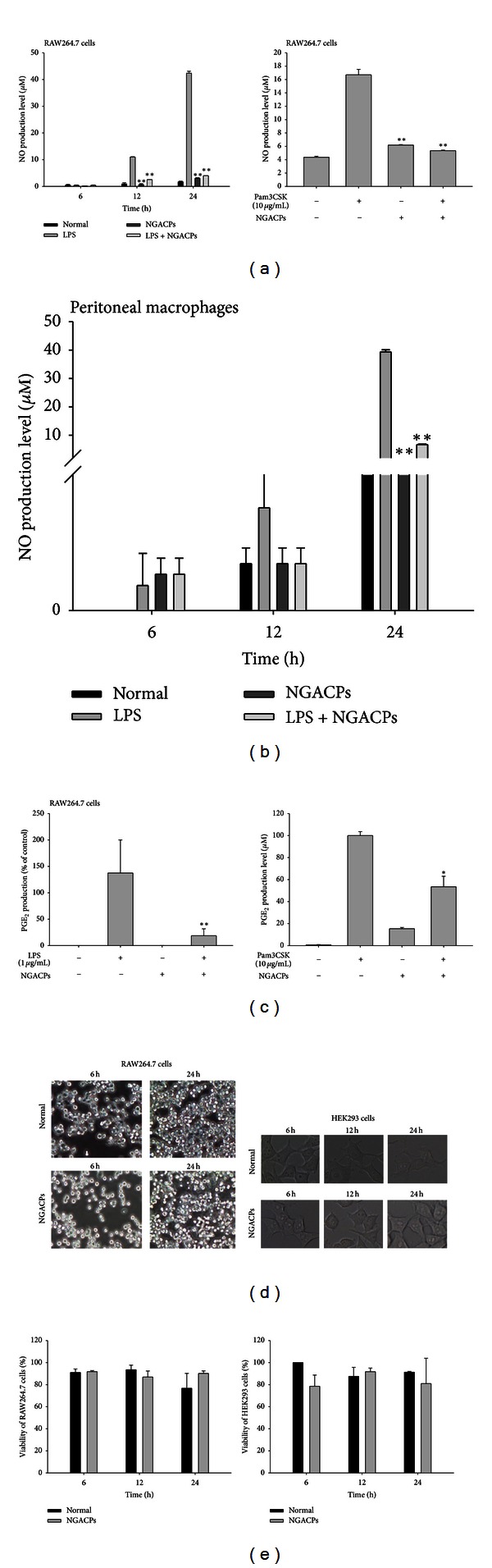
Effect of nanocurrent-generating ACPs (NGACPs) treatment on the production of inflammatory mediators. ((a), (b), and (c)) The levels of NO and PGE_2_ were determined using a Griess assay and EIA with culture supernatants from RAW264.7 cells or peritoneal macrophages that had been treated with NGACP and LPS (1 *μ*g/mL) or pam3CSK (10 *μ*g/mL) for 6 or 24 h (NO and PGE_2_, resp.). (d) Morphological alterations in RAW264.7 cells and HEK293 cells were photographed using a digital camera. (e) The viability of RAW264.7 and HEK293 cells was determined using an MTT assay. *P < 0.05 and **P < 0.01 compared to control.

Data (Figures 1, 2, and 4), expressed as the means ± standard deviation (SD), were calculated from one (*n* = 6) of two independent experiments. Other data are representative of three different experiments with similar results. For statistical comparisons, the results were analyzed using analysis of variance/Scheffe's post hoc test and the Kruskal-Wallis/Mann-Whitney test. All *P*values < 0.05 were considered statistically significant. All of the statistical tests were carried out using the computer program SPSS (SPSS Inc., Chicago, IL, USA). 

## 3. Results and Discussion

It has been reported that electrically generated silver ions increase weak direct currents and are able to inhibit bacterial and fungal growth [21, 22]. Because aluminum and copper are also metals that strongly release electrically generated ions, we first measured the levels of current in a saline solution, RPMI 1640 medium, and cell-cultured medium in the presence or absence of these metals. As Figure 1 shows, both aluminum and copper soaked in these solutions (Figure 1(a)) distinctly increased the nanolevel current from approximately 35 nA to 55 nA (Figure 1(b)). No increase was observed under normal conditions. Therefore, this result strongly suggested that a copper and aluminum patch in cell-plating culture medium could alter the environment for cellular responses by enhancing a nanocurrent. Because electric stimulation has been demonstrated to ameliorate chronic pain and inflammatory symptoms *in vivo *[18–20], we examined whether NGACP treatment could modulate the *in vitro* inflammatory responses of macrophages, and we explored the inhibitory mechanism of NGACP activity.

Interestingly, NGACP exposure strongly suppressed the production of NO from both RAW264.7 cells (Figure 2(a)) and peritoneal macrophages (Figure 2(b)) that had been stimulated with LPS or pam3CSK. Furthermore, NGACP treatment inhibited the release of PGE_2_ under the same conditions (Figure 2(c)), which implied that NGACP treatment could affect both LPS- and pam3CSK-induced inflammatory responses. Analyses of the morphology of RAW264.7 and HEK293 cells (Figure 2(d)) and tests of their viability (Figure 2(e)) clearly demonstrated that NGACP treatment was not cytotoxic to these cells, which indicated that the NO-inhibitory activity of the NGACPs was not caused by nonspecific cytotoxicity.

**Figure 3:**
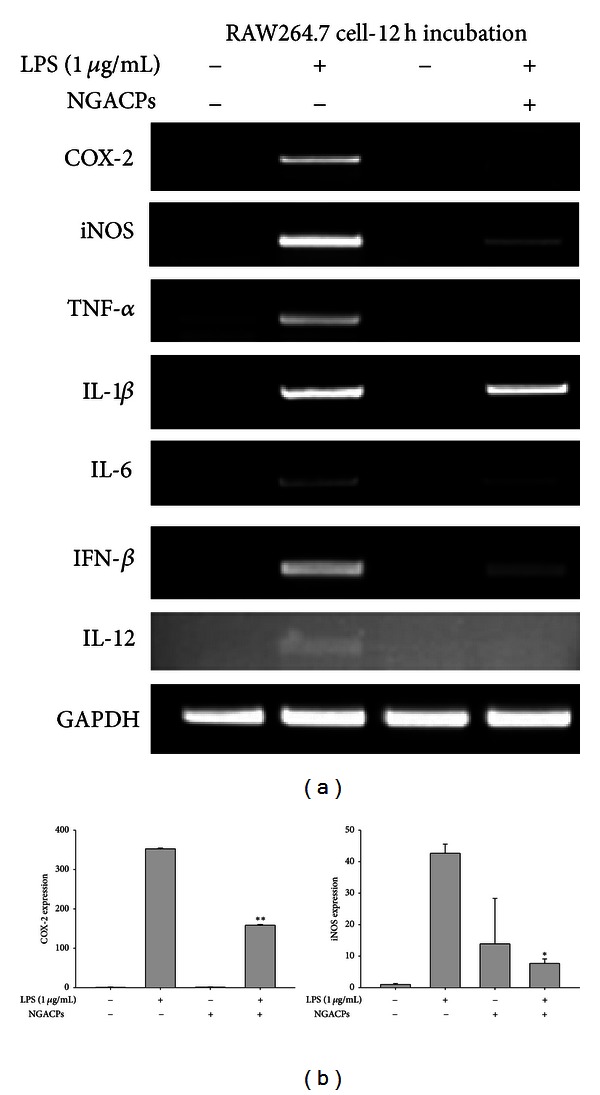
Effect of NGACP treatment on the expression of proinflammatory mRNA. ((a) and (b)) The mRNA levels of proinflammatory genes (COX-2, iNOS, TNF-*α*, IL-1*β*, IL-6, IFN-*β*, and IL-12) were determined using semiquantitative RT-PCR or real-time PCR. *P < 0.05 and **P < 0.01 compared to control.

**Figure 4:**
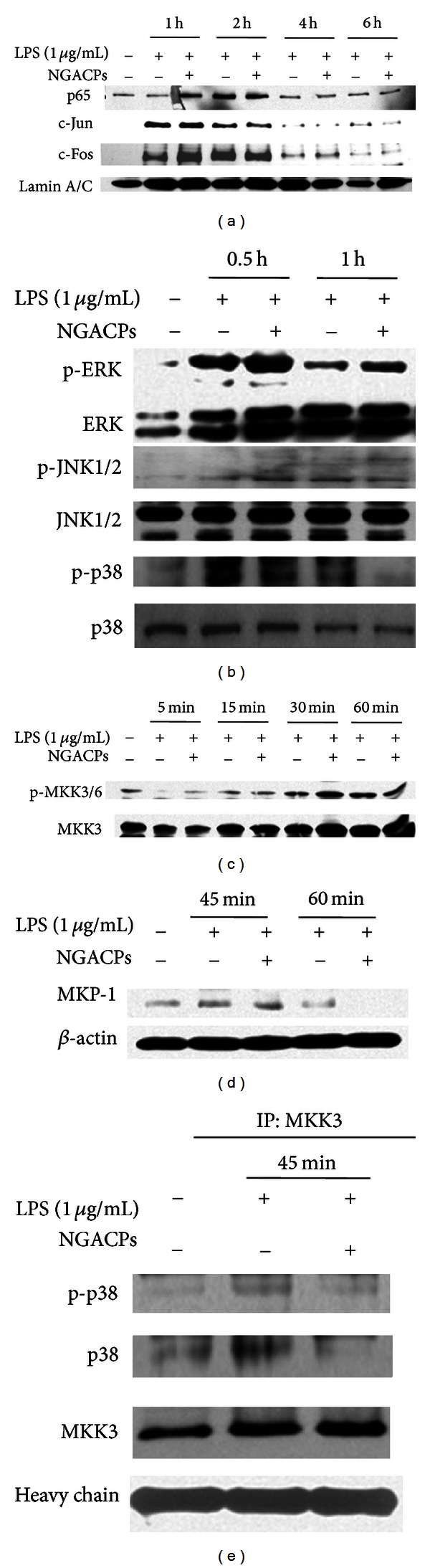
Effect of NGACP on the activation of transcription factors and their upstream signaling events. (a) The levels of transcription factors (p65, c-Jun, and c-Fos) in the nuclear fraction were determined using Western blot analysis of antibodies against total protein. ((b), (c), and (d)) Phosphoprotein or total protein levels of ERK, p38, JNK, MKK3/6, MKP-1, and *β*-actin from cell lysates were determined using Western blot analysis of phospho-specific or total protein antibodies. (e) The molecular complex formation between MKK3 and p38 or p-p38 was evaluated by immunoprecipitation and immunoblotting analysis.

**Figure 5:**
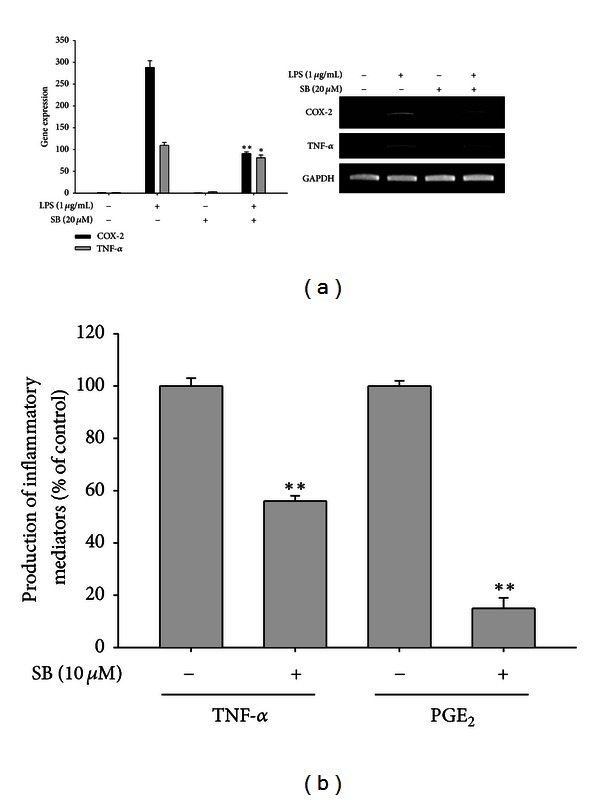
Effect of SB203580 on the expression of COX-2 and TNF-*α* mRNA and the production of TNF-*α* and PGE_2_. ((a) and (b)) The levels of COX-2 and TNF-*α* mRNA were determined using semiquantitative RT-PCR or real-time PCR. (a) The levels of TNF-*α* and PGE_2_ were determined using ELISA and EIA on culture supernatants from RAW264.7 cells that had been treated with SB203580 (20 *μ*M) and LPS (1 *μ*g/mL) for 6 h (TNF-*α*) or 24 h (PGE_2_) h. *P < 0.05 and **P < 0.01 compared to control.

To confirm the inhibitory effect on NO and PGE_2_ production, we first assessed inhibition at the transcriptional level. The mRNA levels of various inflammatory genes such as COX-2, iNOS, TNF-*α*, IL-1*β*, IL-6, IFN-*β*, and IL-12 were measured under NGACP treatment conditions. As Figure 3(a) shows, expression of most of the inflammatory genes except IL-1*β* was suppressed by NGACP treatment. Furthermore, real-time RT-PCR analysis showed similar inhibitory patterns on the expression of iNOS and COX-2 (Figure 3(b)), which implied that transcriptional activation of LPS-induced inflammatory responses was targeted by NGACP treatment. Because transcriptional activation is regulated by the nuclear translocation of various transcription factors such as NF-*κ*B, AP-1, CREB, and STAT-1 [35], the nuclear levels of these proteins were then examined. As Figure 4(a) confirms, NGACP treatment clearly blocked the nuclear translocation of c-Jun at 6 h, while the other transcription factors were also inhibited based on the results for lamin A/C. In addition, AP-1-mediated luciferase activity was also suppressed by NGACP (data not shown). Because c-Jun is a representative transcription factor that is regulated by ERK, JNK, and p38 [36], we examined whether the activation of these enzymes could be blocked by NGACP treatment through analysis of their phosphorylation levels. As Figure 4(b) reveals, the phosphorylation of p38 at 1 h was strongly inhibited by NGACP treatment. In particular, because phosphorylation of MKK3/6, an upstream enzyme that phosphorylates p38 [37], was not blocked (Figure 4(c)) and the protein level of phospho-p38 diphosphatase, MKP-1 [38], was not enhanced (Figure 4(d)), NGACP treatment seems to directly target MKK3/6, although a specific enzyme assay would be required to obtain better evidence. In agreement with this finding, signaling complex formation between p38 and MKK3 was also defective, as demonstrated through immunoprecipitation and immunoblotting analyses (Figure 4(e)). Therefore, these results strongly suggest that the MKK3/p38 pathway could be the major inhibitory target of NGACP treatment. Finally, we confirmed the functional significance of p38 in the inflammatory response by using a specific inhibitor, SB203580 (SB), on LPS-activated RAW264.7 cells. SB also strongly suppressed the expression of COX-2 and TNF-*α* mRNA, as assessed using RT- and real-time PCR (Figure 5(a)) and inhibited the release of TNF-*α* and PGE_2_ (Figure 5(b)), which is in agreement with previously reported results [39]. 

The mechanism through which NGACP treatment can affect the p38-mediated AP-1 activation signaling cascade is not yet clear. Similar to our results (Figure 4(b)), it has been reported that Zn/Cu-derived microcurrents are able to suppress NF-*κ*B via crosstalk with p38 in primary keratinocytes that have been stimulated with TNF-*α* [40]. In contrast, under normal conditions, electrical stimulation increased the phosphorylation of p38 in keratinocytes and human mesenchymal stromal cells [40, 41], which implies that inhibition of p38 signaling could be a stimulus-dependent phenomenon. Thus, NGACP treatment only suppressed the phosphorylation of p38 that was induced by treatment with LPS as a TLR4 ligand [42] and inhibited NO and PGE_2_ production during exposure to LPS and pam3CSK, which is a TLR2 ligand [42]. Furthermore, electrical stimulation has been shown to activate or inhibit the activities of other enzymes such as PI3K and AKT in muscle and liver cells [43, 44], ERK in human mesenchymal stromal cells [41], and JNK1/2 in the injured kidneys of Alport mice [45]. In the case of p38 activation, it has been found that hydrogen peroxide that was produced by a Zn/Cu-triggered microcurrent plays a critical role in phosphorylation [40]. However, the inhibition of LPS-induced p38 activation does not appear to be explained by this event. The fact that NGACP treatment did not only increase MKP-1 levels to dephosphorylate p38 (Figure 4(d)) but also suppressed the activation of p38-phosphorylating MKK3/6 (Figure 4(c)) strongly suggests that there is an indirect inhibitory mode of action that suppresses the p38/c-Jun pathway. Further studies to get insights will be followed in the next experiments.

In summary, we have found that NGACP treatment is able to suppress LPS-induced inflammatory responses such as NO and PGE_2_ production in both RAW264.7 cells and peritoneal macrophages at the transcriptional level. According to our immunoblotting and immunoprecipitation analyses, p38/AP-1 could be the major inhibitory pathway in the NGACP-mediated anti-inflammatory response. Therefore, these results strongly suggest that NGACP treatment could be applied to the treatment of various inflammatory diseases such as arthritis and colitis. To further test this possibility, additional relevant *in vivo* efficacy tests will be conducted in the future.

## Figures and Tables

**Table 1:** Sequences of primers used in semiquantitative and real-time PCR analyses.

Name		Sequence (5′ to 3′)
Real-time PCR		

iNOS	F	GGA GCC TTT AGA CCT CAA CAG A
	R	TGA ACG AGG AGG GTG GTG
COX-2	F	GGGAGTCTGGAACATTGTGA
	R	GCACATTGTAAGTAGGTGGA
TNF-*α*	F	TGC CTA TGT CTC AGC CTC TT
	R	GAG GCC ATT TGG GAA CTT CT
GAPDH	F	CAA TGA ATA CGG CTA CAG CAA C
	R	AGG GAG ATG CTC AGT GTT GG

Semiquantitative RT-PCR		

TNF-*α*	F	TTGACCTCAGCGCTGAGTTG
	R	CCTGTAGCCCACGTCGTAGC
IL-1*β*	F	CAGGATGAGGACATGAGCACC
	R	CTCTGCAGACTCAAACTCCAC
IL-6	F	GTACTCCAGAAGACCAGAGG
	R	TGCTGGTGACAACCACGGCC
COX-2	F	CACTACATCCTGACCCACTT
	R	ATGCTCCTGCTTGAGTATGT
iNOS	F	CCCTTCCGAAGTTTCTGGCAGCAGC
	R	GGCTGTCAGAGCCTCGTGGCTTTGG
IFN-*β*	F	CCACCACAGCCCTCTCCATCAACTAT
	R	CAAGTGGAGAGCAGTTGAGGACATC
IL-12	F	CAGAAGCTAACCATCTCCTGGTTTG
	R	TCCGGAGTAATTTGGTGCTTCACAC
GAPDH	F	CACTCACGGCAAATTCAACGGCAC
	R	GACTCCACGACATACTCAGCAC

F: forward, R: reverse.
